# End-stage care for children after heart transplant

**DOI:** 10.3389/frtra.2023.1221166

**Published:** 2023-06-27

**Authors:** Melanie D. Everitt

**Affiliations:** Department of Pediatrics, Children’s Hospital Colorado, University of Colorado, Aurora, CO, United States

**Keywords:** heart tranplantation, pediatic, chronic graft dysfunction, retransplant, chronic kidney disease

## Abstract

Heart transplant is performed annually in over 600 children worldwide to treat life-limiting cardiac disease. Conversations regarding waitlist mortality, post-transplant morbidity and mortality, and goals of care are commonplace pre-transplant. However, there is a void of information and resources for providers and families when end-stage disease recurs in the long-term transplant recipient. The purpose of this review is to discuss the care of the pediatric heart transplant recipient with chronic cardiac dysfunction occurring years after a successful transplant. This includes a need for transplant providers to have education and training related both to palliative care and medical ethics to improve shared decision making with patients and families.

## Introduction

**Figure 1:**
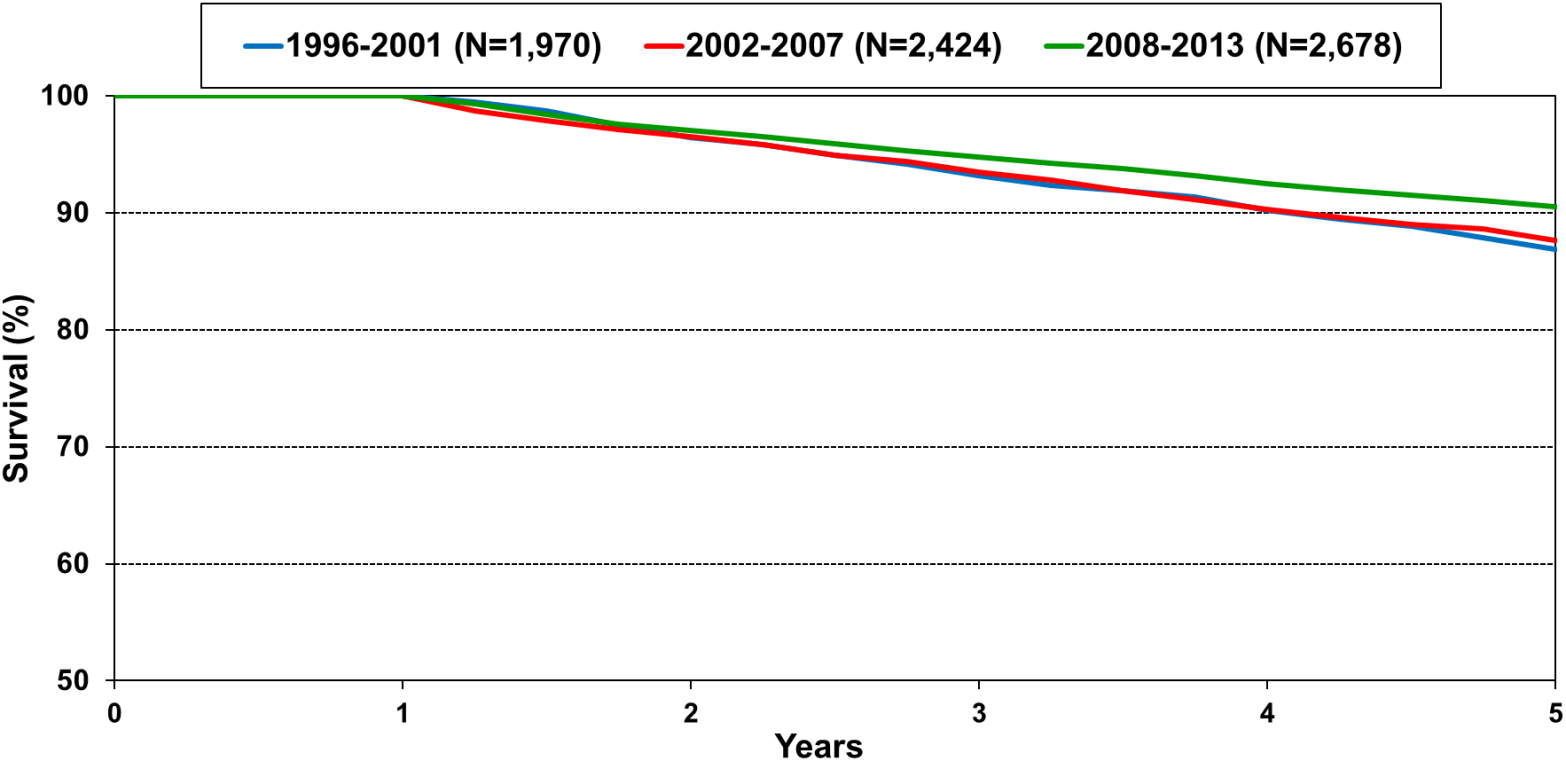
Kaplan-meier survival within 5 years conditional on survival to 1 year shown by era for pediatric heart transplant recipients in the International Society for Heart and Lung Transplantation registry. Note the *y*-axis is truncated. Pairwise comparison between the recent era 2008–2013 is significantly different between 1996 and 2001 and 2002–2007 at *p*-value <0.05 (1).

The number of pediatric heart transplants performed annually has steadily increased, and transplant survival has also improved (1). Survival within 5 years conditional on 1-year survival is 90% in the most recent era of transplant (Figure 1) (1). With more children undergoing heart transplant and recipients surviving longer, the number of children living with a transplanted heart has grown exponentially. Thus, it is important to be knowledgeable in the care of the long-term transplant recipient. This includes evaluation and care of chronic graft dysfunction or other severe non-cardiac morbidities.

### Survival and chronic graft dysfunction

For those who survive at least 5 years post heart transplant, there is a steady decline in survival over the ensuing 2 decades with estimated survival 73% at 15 years and 51% survival at 25 years (2). The number of children alive but with the sequelae of chronic graft dysfunction has not been described and may be more prevalent than expected. A paper by Kindel et al. focusing on cardiac allograft vasculopathy (CAV) describes features of cardiac dysfunction at the time of coronary assessments (3). In this multi-institutional analysis of 8,000 hemodynamic assessments in 2,000 pediatric heart transplant recipients, the left ventricular ejection fraction (LVEF) was depressed (<45%) in 3.5%, right atrial pressure was elevated (>13 mmHg) in 4.4%, and pulmonary capillary wedge pressure was elevated (>15 mmHg) in 6.4% of assessments. Graft survival was worse with any degree of graft dysfunction and CAV compared to those with CAV but normal graft function (3). Unfortunately, this study did not assess outcomes of chronic graft dysfunction in the absence of CAV, but it is expected that these outcomes would also be poor.

### Retransplantation

While some children with end-stage disease after heart transplant are eligible for retransplant, others may choose not to undergo retransplant or are not eligible related. Contraindications to retransplant are like those put forth for primary transplant candidacy (4). Multiple large registry studies have shown lower survival after retransplant compared to primary transplant (5–7). A recent study by Conway et al. found similar early complications after heart transplant. However, retransplant patients had a higher risk of early and late rejection, CAV, earlier time to graft failure, and higher risk for late renal dysfunction (6). In multivariate analysis, retransplant was a risk factor for death at 1, 5, 10, and 15 years after second transplant compared to primary transplant (6). These data should be considered when discussing retransplant as an option for end stage care with patients and families.

### Medical therapy

**Figure 2:**
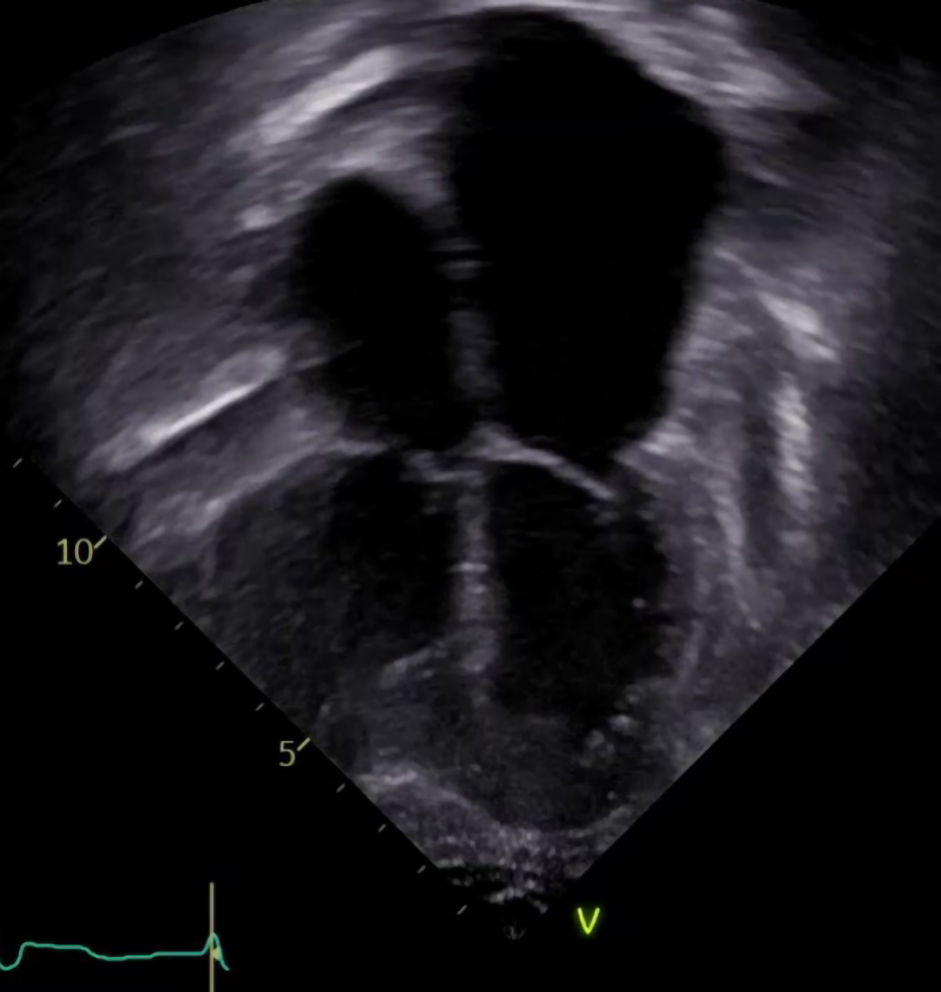
The echocardiographic image illustrates the typical features of chronic graft dysfunction in a child 16 years post heart transplant with CAV and diastolic heart failure. There is marked dilation of the atria, dilation of the pulmonary veins, and normal left ventricular size and function. This patient is 7 years after severe CAV requiring stenting of a focal narrowing of the left anterior descending artery. He remains alive with oral anti-congestive therapies to treat symptoms of his chronic graft dysfunction.

Most commonly transplant graft dysfunction is due to cardiac allograft vasculopathy and is a progressive and diffuse process affecting multiple vessels. Systolic function is generally preserved early in the disease course, and diastolic heart failure manifests from restrictive physiology of both the right and left ventricles (Figure 2). Transplant graft dysfunction is challenging to manage due to the nature of the disease (heart failure with preserved ejection fraction) and to cardiorenal syndrome concomitant with kidney disease from long-term calcineurin use. There are few studies to guide heart failure management in children with cardiac disease of their native heart, and there are no studies to guide heart failure management of the failing transplanted heart with or without CAV. Like the approach taken in the pretransplant management of pediatric heart failure, medications to ameliorate symptoms followed by medications associated with improved survival in adult nonischemic and ischemic heart disease are the foundations of care (8).

The care of these patients becomes increasingly difficult as the cardiac and renal disease progresses. Most pediatric cardiologists have experience with using diuretics, renin-angiotensin-aldosterone blockade, beta blockade, and digoxin to treat pediatric heart failure. However, the end-stage transplant patient may benefit from different or newer agents where experience in children is less widespread. Tolvaptan, neprilysin inhibitors, sodium-glucose cotransporter-2 (SGLT2) inhibitors, and ivabradine have all been utilized at our center with varying effects to treat refractory heart failure symptoms. In general, oral medications were utilized to achieve patient-centered goals such as limiting intravenous therapies and recurrent hospitalizations. The effect on survival is not known. Without studies to guide therapy or large series of pediatric heart transplant patients receiving these treatments, specific recommendations cannot be made. Suffice it to say, it is reasonable to consider newer or alternative heart failure medications in children with chronic graft dysfunction who have refractory symptoms, intolerance to other anti-congestive therapies, or where benefit may be derived from other effects of the treatment such as the potential for SGLT2 inhibitors to improve serum glucose. The tenets of safe prescribing include a review of drug-drug interactions, initiation of the lowest dose possible with titration to effect, knowledge of side effects, and monitoring for adverse effects including renal impairment, liver injury, and infection.

An additional focus of end-stage care is an assessment of what medications the patient is willing and able to take, including modification of immune suppression in some cases. This assessment of medication burden is different from a decision by a patient to discontinue anti-rejection medication as a part of end-of-life choices. On the contrary, the focus is increased adherence to therapies with acute benefit. This can be accomplished through review of the patient’s medical regimen to identify once-daily alternatives, emphasizing medications that alleviate symptoms or have a proven benefit, and limiting medications that are contributing to renal failure and further exacerbates heart failure. To decrease pill burden or side effects of immune suppression in end-stage patients, our center has transitioned patients to either extended release tacrolimus or monthly basiliximab. We have described our use with monthly basiliximab in end stage patients to limit further calcineurin inhibitor exposure in children and to decrease recurrent admissions for renal failure or rejection. Ten recipients who were a median of 10 years post-transplant and ineligible for retransplant received basiliximab for a median of 5.5 monthly doses. Mortality was high in this group of children with end-stage disease (70%) and breakthrough rejections did occur in 3 patients (9). This therapy is costly and requires intravenous infusion in a medical setting. The benefit of continuation should be reassessed at regular intervals.

### Shared decision making and the value of palliative care

Shared decision making is an important component in the care of adults with advanced heart failure, and many aspects of this are relevant to pediatric heart transplant recipients with end-stage disease. Shared decision making involves fully informing patients and families of the risks and benefits of multiple reasonable therapeutic approaches and making patient-centered choices related to their individual values, preferences, and the likelihood of beneficence of an intervention. Enabling patients to manage heart failure through self-care tools at home may align with their goals of care. Since worsening heart failure signs/symptoms can indicate either acute rejection or progressive graft dysfunction without acute rejection, medical teams can be reluctant to allow “self-care” and adjustment of diuretics or fluid intake at home. However, if the patient’s goals are to reduce the burden of urgent visits, invasive testing, and unnecessary hospitalizations, then there may be a role for individualized “self-care” of heart failure symptoms. Tools for this can be modified from adult heart failure resources and individualized to the patient’s age and clinical condition (10). A review of prognosis and treatment preferences at the time of identifiable events that portend a worse prognosis is also encouraged in advanced heart failure and can be adapted for discussions after events in pediatric heart transplant that portend a worse prognosis. Examples include a rejection episode with severe hemodynamic compromise or the presence of CAV and graft dysfunction (3, 11).

Studies examining end of life care for children after heart transplant have found a high prevalence of intensive care therapies and invasive life-supporting interventions prior to death. In a Pediatric Heart Transplant Society analysis of end-of-life care in pediatric heart transplant recipients, 22% of post-transplant deaths occurred in the hospital setting. Of these, 74% were in the intensive care unit, 52% receiving mechanical ventilation, and 18% supported by mechanical circulatory devices prior to death (12). The authors note the absence of information pertaining to palliative care services, advanced directives, and resuscitative limitations in this registry. In a single center study by Hollander et al, end-of-life care was described in 23 pediatric heart transplant recipients (13). Less than one-third of patients had a palliative care consult performed prior to death despite 88% of children in the intensive care unit, 74% intubated, 30% on mechanical circulatory support, and 22% receiving dialysis at end-of-life. At the time of death, only 13% of children had limitations to resuscitative care noted in their chart. The circumstance of death was compassionate extubation or ECMO decannulation in over half of deaths (13). In a review of 26 deaths during a 10-year period at our center, there were 13 children whose death was not unexpected based on chronicity and severity of illness. Of these, 12 had a palliative care consult and half of these had transitioned to hospice with concurrent care by the transplant team. These data highlight the challenging circumstances surrounding end of life care of the transplant recipient. While these discussions can be aided by palliative care providers and medical ethics experts, the need for palliative services often outpaces the resources available in the outpatient and inpatient setting. Thus, it is imperative for pediatric transplant professionals to gain knowledge and feel comfortable engaging in these conversations with their patients and multi-disciplinary teams.

### Multi-disciplinary transplant team as primary palliative care provider

A recent American Heart Association Scientific Statement provides a useful review of palliative care for children with heart disease (14). The principles and strategies put forth are applicable to end-stage care for children after heart transplant. The statement distinguishes between the practice of palliative care by the child’s primary team and that of specialized palliative care resources. This distinction is important because specialized resources may not be available or may be limited to inpatient needs. Additionally, it is the primary transplant team who is at the forefront of delivering care when events occur that change quality of life or alter life expectancy of the transplant patient. When palliative care specialists are consulted, their expertise augments, rather than replaces, the care delivered by the primary transplant team.

The importance of a multi-disciplinary heart transplant team in providing primary palliative care cannot be overstated. The well-established integration of team members enables clear and timely communication which is essential in guiding patients and their families through difficult conversations. The role of the long-term transplant provider is to openly discuss disease prognosis and treatment and to identify gaps in the patient/family’s understanding of the disease progression (14). Medical team members can help the child/family understand if recovery to the child’s prior state of well-being is expected after an episode of severe rejection, post-transplant malignancy, stroke, or life-threatening infection. Neuropsychologists play a role in learning how best to present information that is difficult and assessing the patient’s capacity for shared decision-making. Social workers can gain insight and provide resources specific to end-of-life care such as financial stressors related to parental time off work, separation from home and local community, sibling support, and coping strategies. Untreated mental illness in the child can limit the medical team’s ability to discern or mitigate symptoms of end-stage heart failure. Dyspnea, sleep disturbances, palpitations, loss of appetite, and fatigue may be due to mental health issues or declining physical health. Psychologists aid in diagnosing and treating mood disturbances and mental illness in the transplant patient. Lastly, the transplant pharmacist can provide useful information related to symptom management and drug interactions.

Not to be overlooked is how end-of-life care and patient death impacts the transplant team members themselves. Multiple studies of healthcare workers have identified lack of preparedness and insufficient coping skills to deal with the negative feelings associated with the death of a child. This lack of coping contributes to lower work satisfaction, burn out, secondary stress disorder, and compassion fatigue in medical providers (15–17). While there are no studies specifically addressing this among pediatric heart transplant team members, it is likely that transplant teams can benefit from strategies utilized in other areas of healthcare. These include peer support groups, institutional protocols for dealing with death, and educational opportunities related to coping skills including recognizing the difference between empathy vs. sympathy with respect to showing compassion without internalizing the family’s pain and suffering (15, 18).

## Discussion

This review focuses on the recurrence of end stage heart failure years after a successful transplant in childhood. It addresses the need to understand the patient’s and family’s goals of care, including whether retransplant is desirable. Palliative care specialists are valuable for these discussions but are not always available to patients due to limited resources and the timing of crucial conversations. Heart transplant providers should be knowledgeable and skilful in engaging in conversations related to end stage care. Transplant providers may need to be open to alternative means of managing patients with end stage heart failure after transplant when the patient’s goal of care is to limit invasive testing, hospitalizations, or urgent visits. Laying the foundation for these conversations as early as possible and building upon pre-transplant conversations are key to understanding the goals of care as they change over the life of the transplant recipient. Patients, families, and team members benefit when goals of care discussions are woven throughout the continuum of care after transplant. Team members further benefit from peer support and time to share their feelings and thoughts after death of a patient.

## Data Availability

The original contributions presented in the study are included in the article, further inquiries can be directed to the corresponding author.

## References

[1] Singh TP Cherikh WS Hsich E Chambers DC Harhay MO Hayes D Jr International society for heart and lung transplantation. The international thoracic organ transplant registry of the international society for heart and lung transplantation: twenty-fourth pediatric heart transplantation report—2021; focus on recipient characteristics. J Heart Lung Transplant. (2021) 40(10):1050–9. 10.1016/j.healun.2021.07.022 34420853 PMC10281816

[2] Rossano JW Singh TP Cherikh WS Chambers DC Harhay MO Hayes D Jr International society for heart and lung transplantation. The international thoracic organ transplant registry of the international society for heart and lung transplantation: twenty-second pediatric heart transplantation report—2019; focus theme: donor and recipient size match. J Heart Lung Transplant. (2019) 38(10):1028–41. 10.1016/j.healun.2019.08.002 31548029 PMC6819143

[3] Kindel SJ Law YM Chin C Burch M Kirklin JK Naftel DC Improved detection of cardiac allograft vasculopathy: a multi-institutional analysis of functional parameters in pediatric heart transplant recipients. J Am Coll Cardio. (2015) 66(5):547–55. 10.1016/j.jacc.2015.05.063 26227194

[4] Mehra MR Canter CE Hannan MM Semigran MJ Uber PA Baran DA International society for heart lung transplantation (ISHLT) infectious diseases, pediatric and heart failure and transplantation councils. The 2016 international society for heart lung transplantation listing criteria for heart transplantation: a 10-year update. J Heart Lung Transplant. (2016) 35(1):1–23. 10.1016/j.healun.2015.10.023 26776864

[5] Mahle WT Vincent RN Kanter KR. Cardiac retransplantation in childhood: analysis of data from the united network for organ sharing. J Thorac Cardiovasc Surg. (2005) 130(2):542–6. 10.1016/j.jtcvs.2005.02.050 16077425

[6] Conway J Manlhiot C Kirk R Edwards LB McCrindle BW Dipchand AI. Mortality and morbidity after retransplantation after primary heart transplant in childhood: an analysis from the registry of the international society for heart and lung transplantation. J Heart Lung Transplant. (2014) 33(3):241–51. 10.1016/j.healun.2013.11.006 24462559

[7] Chin C Naftel D Pahl E Shankel T Clark ML Gamberg P Cardiac re-transplantation in pediatrics: a multi-institutional study. J Heart Lung Transplant. (2006) 25:1420–4. 10.1016/j.healun.2006.09.020 17178335

[8] Heidenreich PA Bozkurt B Aguilar D Allen LA Byun JJ Colvin MM 2022 AHA/ACC/HFSA guideline for the management of heart failure: a report of the American college of cardiology/American heart association joint committee on clinical practice guidelines. Circulation. (2022) 145:e895–1032. 10.1161/CIR.0000000000001063 35363499

[9] Chen TT Greene MM Everitt MD Simpson KE. Basiliximab as maintenance immunosuppression in heart transplant recipients: a single pediatric center experience. Pediatr Transplant. (2023) 27(2):e14438. 10.1111/petr.14438 36397270

[10] American Heart Association. Get With the Guidelines. (2022). Available at: https://www.heart.org/-/media/Files/Professional/Quality-Improvement/Get-With-The-Guidelines-HF/Educational-Materials/DS18660 (Accessed May 8, 2023).

[11] Kleinmahon JA Gralla J Kirk R Auerbach SR Henderson HT Wallis GA Cardiac allograft vasculopathy and graft failure in pediatric heart transplant recipients after rejection with severe hemodynamic compromise. J Heart Lung Transplant. (2019) 38(3):277–84. 10.1016/j.healun.2018.12.011 30638837

[12] Cousino MK Yu S Blume ED Henderson HT Hollander SA Khan S Circumstances surrounding end-of-life in pediatric patients pre- and post-heart transplant: a report from the pediatric heart transplant society. Pediatr Transplant. (2022) 26(2):e14196. 10.1111/petr.14196 34820983 PMC10466174

[13] Hollander SA Chen S Luikart H Burge M Hollander AM Rosenthal DN Quality of life and metrics of achievement in long-term adult survivors of pediatric heart transplant. Pediatr Transplant. (2015) 9(1):76–81. 10.1111/petr.12384 25388808

[14] Blume ED Kirsch R Cousino MK Walter JK Steiner JM Miller TA American heart association pediatric heart failure and transplantation committee of the council on lifelong congenital heart disease and heart health in the young. Palliative care across the life span for children with heart disease: a scientific statement from the American heart association. Circ Cardiovasc Qual Outcomes. (2023) 16(2):e000114. 10.1161/HCQ.0000000000000114 36633003 PMC10472747

[15] Pradas-Hernández L Ariza T Gómez-Urquiza JL Albendín-García L De la Fuente EI Cañadas-De la Fuente GA. Prevalence of burnout in paediatric nurses: a systematic review and meta-analysis. PLoS One. (2018) 13(4):e0195039. 10.1371/journal.pone.0195039 29694375 PMC5918642

[16] Carreño MA Yago AM Bellón JJ Baeza-Mirete M Muñoz-Rubio GM Rojo Rojo A. An exploratory study of ICU pediatric nurses’ feelings and coping strategies after experiencing children death. Healthcare (Basel). (2023) 11(10):1460. 10.3390/healthcare11101460 37239746 PMC10218027

[17] Rodríguez-Rey R Palacios A Alonso-Tapia J Pérez E Álvarez E Coca A Burnout and posttraumatic stress in paediatric critical care personnel: prediction from resilience and coping styles. Aust Crit Care. (2019) 32(1):46–53. 10.1016/j.aucc.2018.02.003 29605169

[18] Granek L Barrera M Scheinemann K Bartels U. When a child dies: pediatric oncologists’ follow-up practices with families after the death of their child. Psychooncology. (2015) 24(12):1626–31. 10.1002/pon.3770 25707675

